# Gender differences in the interaction effect of cumulative risk and problem-focused coping on depression among adult employees

**DOI:** 10.1371/journal.pone.0226036

**Published:** 2019-12-16

**Authors:** Shi-Min Chen, Pei-Zhen Sun

**Affiliations:** 1 School of Public Administration, China University of Mining and Technology, Xuzhou, China; 2 School of Educational Science, Jiangsu Normal University, Xuzhou, China; Middlesex University, UNITED KINGDOM

## Abstract

The adult employees suffer from various pressure and their mental health has been paid more and more attention to. This study has two purposes, namely, (1) to investigate the gender differences in the stressors and utilization frequency of problem-focused coping among adult employees and (2) to explore the gender differences in the interaction effect of cumulative risk and problem-focused coping on depression among adult employees. The cumulative risk of employees was assessed in the following six ways: health pressure, family economic pressure, love and marriage problems, conflicts among family members, work stress and friend support. Problem-focused coping was measured by the three dimensions of active coping, planning, and using instrumental support from the Brief COPE scale, and depression was assessed by the Self-rating Depression Scale. The participants consisted of 406 Chinese employees. The results showed that (1) the cumulative risk of male employees was marginally significantly higher than that of female employees; (2) there was no significant difference in the utilization frequency of problem-focused coping between male and female employees; and (3) problem-focused coping moderated the relationship between cumulative risk and depression for male employees but not for female employees. This study indicates that problem-focused coping has a stronger effect on depression for male employees than for female employees.

## Introduction

Depression is a major public health problem throughout the world and is characterized by lowered mood, loss of capacity to experience pleasure, an increased sense of worthlessness, fatigue, and preoccupation with death and suicide [1]. Epidemiological research on the prevalence and incidence of depressive symptoms and unipolar depressive disorders has consistently shown a preponderance in women compared with men [2–4]. Studies from the developmental perspective show that there is no gender difference among boys and girls in childhood. Meta-analysis indicated that the odds ratio of depressive symptoms among women to men (OR) at ages 8–12 years was 1.18 with Cohen’s d = 0.09 [5]. The gender differences of depression emerge at ages 13–15 years (OR = 1.89, d = 0.35), peak at ages 16–19 years (OR = 2.10, d = 0.41), then decline in the 20s and stay relatively stable afterwards at roughly OR = 1.50 (d = 0.20) [5]. However, there has been little research on the gender differences in depression among adult employees. This study intends to further explore the gender differences in depression (1) by using cumulative risk instead of a single risk factor to evaluate the stressors and (2) by examining the gender differences in the interaction effect of stressors and problem-focused coping on depression among adult employees.

### Cumulative risk

Multiple risks theory holds that individuals often suffer multiple risk factors simultaneously [6, 7]. Different risk factors do not work independently but often co-occur. That is, there is a chain reaction among negative events. For example, suffering from serious disease is likely to cause economic pressure and interpersonal conflict. Therefore, focusing solely on a single or a few risk factors is not consistent with the reality of most individuals. Furthermore, individuals are often not sensitive to single risk factors. Only when there are many risk factors does an individual face the possibility of injury [6, 7].

There are different approaches to measure multiple risks. The usual approach of assessing multiple risks is to form a composite index by combining the different risk factors into one summary score. Compared with summary score, cumulative risk models of multiple risk exposure define risk factors dichotomously (risk exposure = 1, no risk exposure = 0). Cumulative risk index is operationalized by summing across different multiple dichotomous risk factors. Evans, Li and Whipple (2013) compared cumulative risk to other approaches to multiple risk factor assessment among 140 studies and analyzed its weaknesses and strengths [6]. Although the cumulative risk model has some shortcomings, e.g., it loses information when converting continuous risk factors into a dichotomy, it also has some outstanding advantages. Cumulative risk captures the type of risk factor exposure that truly matters in the undesirable outcomes of an individual, and a cumulative risk metric is parsimonious. The severity of risk exposure that one suffers from can be known straightforwardly according to the cumulative risk index; however, this severity is difficult to know according to the summary score from which we often do not know how many scores mean a high risk exposure. For example, we use 10 scales to measure the risk exposure. If one’s summary score is 100, it is often difficult for us to know the types and severity of the risks that he/she is exposed to. However, if his/her cumulative risk index is 4, we can know easily that he/she is suffering from four types of high risk exposure. Therefore, cumulative risk has been used to assess the stressor more and more [8–12].

### Explanation for the gender differences in depression

Several explanations have been proposed to account for the gender differences in depression. First, women are more vulnerable to violence including physical abuse and sexual abuse. Child maltreatment has devastating consequences on many domains of child development, both in the short and the long term, which causes more depression in women [13]. More fear of violence in the neighbourhood results in higher depression among emerging female adults than among emerging male adults [14]. Moreover, gender inequality is linked to discrimination against women. Women receive less resources and lower social status, which results in their feelings of inferiority and depression [5].

The second explanation is gender role socialization theory [15, 16]. During the process of socialization, men are expected to be capable and make achievements. They are encouraged to be independent and self-reliant, which forms the self-construal of agency. However, women are expected to be gentle and to care for other people. They are encouraged to be connected to other people and have concern for their feelings, which forms the self-construal of communion. Different social expectations and self-construal make men and women responsive to different stressors: men are responsive to agentic stressors that are related to achievement, whereas women are sensitive to communal stressors that are related to relationships. A survey of middle school students showed that girls suffered from more pressures, especially interpersonal pressures [3, 4, 15].

The third viewpoint is presented from the perspective of coping. The theory of sociobiology, which is based on animal studies, shows that men are more inclined to use a "fight or flight" coping style, while women tend to adopt the "tend and befriend" coping style [17]. Gender role socialization theory proposes that society expects men to be independent, aggressive and successful; thus, when they face pressure, they use more problem-focused coping than women [15, 17]. Both the theory of sociobiology and gender role socialization theory indicate that when facing adversity, men employ more problem-focused coping than women employ [15, 17]. When facing a difficult situation, men who are assertive, self-reliant, competitive, willing to take risks, and determined to succeed are more confident in their capacities to address problems. Consequently, they are less likely to become depressed in stressful situations [4].

In addition, all types of biomedical factors such as genetic, hormonal factors and neurotransmitter are also proposed. They have some effect on the gender differences in depression but to a lesser extent than environmental factors [2].

### Purposes of this study

Previous studies mainly focused on the gender differences in depression among adolescents [18–21], emerging adults [14], and older adults [22–26] but paid less attention to adult employees. As is known to all, the adult employees undergo all kinds of pressure including work stress, caring for family members, economic pressure, health problem and so on. Mental health of employees has become a serious problem. Previous surveys showed that the prevalence of depression was up to 40–80% among employees in four metropolitan Chinese cities (Beijing, Shanghai, Guangzhou, Shenzhen) [27–29], 32% among bank employees in Brazil [30], 5.9% among national employees in Korea [31]. Moreover, the gender differences of adult employees have their unique characteristics including stressors and problem-focused coping. In terms of stressors, similar to male employees, female employees also need to make money, solve various problems in their work, and face various agentic stressors. Regarding problem-focused coping, many female employees have obtained higher education. They continually use problem-focused coping strategies in the process of pursuing their academic performance, looking for a job, and finishing their work tasks. It is likely that the gender differences in the stressors and problem-focused coping between male and female employees is decreasing. However, these gender differences have not been empirically examined. Therefore, we present two questions of this study as follows.

**Question 1:** What are the gender differences in the stressors among adult employees?
**Question 2:** What are the gender differences in the utilization frequency of problem-focused coping among adult employees?

Previous studies have indicated that adaptive coping strategies have been shown to at least partially counteract or compensate for the negative effects of depression and poor mental health [32–34]. Since problem-focused coping is an important adaptive coping and cumulative risk is a better index to assess the stressors that affect depression than a single stressor such as family structure [35], obesity [36], and child maltreatment experience [37], we propose the following hypothesis:

**Hypothesis:** Problem-solving coping moderates the effect of cumulative risk on depression.

If the hypothesis is validated, considering the decrease of gender differences in the cumulative risk and problem-focused coping, are there gender differences in the interaction effect of cumulative risk and problem-solving coping on depression? Accordingly, we put forward a third question as follows:

**Question 3:** Are there gender differences in the moderating effect of problem-focused coping on cumulative risks and depression?

## Methods

### Participants

The participants were from 6 organizations in Guangzhou and Shenzhen, two metropolises in China. Validation questions were used to eliminate participant data in case of inconsistent responses to lie-detection items (“I often smile” and “I am always poker-faced”). A total of 406 valid questionnaires were obtained from 236 males and 170 females who ranged in age from 18 to 42 years with a mean age of 25.9 years (SD = 5.1). The participants comprised 89 technical secondary school graduates, 101 junior college graduates, 208 undergraduates, and 8 master’s degree graduates. In total, 359 were junior employees, 47 were mid-level employees, and 3 were senior employees. Twenty-seven were human resource employees, 3 were financial and accounting employees, 137 were salespersons, 4 were purchasing employees, 168 were engineers and technicians, and 67 were logistical employees.

According to Shieh (2009) [38], when the significance level α was set to 0.05, the power was set to 0.90, and the correlational efficient between the predictor and moderator was 0.50, the minimum sample size was 137; when the significance level α was set to 0.05, the power was set to 0.90, and the correlational efficient between the predictor and moderator was 0.10, the minimum sample size was 169. The participants consisted of 406 employees (236 males and 170 females) in this study. It could be seen that the sample sizes of the male and female employee in this study were enough for moderating effect test.

### Procedure

The procedure included the following steps. We obtained permission from the management of all organizations involved and ethical approval from the Research Ethics Committee of the Research Ethics Committee of China University of Mining and Technology (NO.: 201803005). Then, the questionnaires were delivered to designated coordinators of data collection, and the coordinators distributed the questionnaires before meetings or group trainings. The participants were reminded that their participation in the study was voluntary and that they could discontinue their participation in the study at any time. They were also assured that their responses would be kept confidential. They were asked to provide written informed consent after the procedures had been fully explained.

### Measures

#### Cumulative risk of employees

The cumulative risk of employees has not been evaluated in previous studies, but in this study, it was assessed in six aspects, namely, health pressure, family economic pressure, love and marriage problems, conflicts among family members, work stress, and friend support. Because many participants are reluctant to report their child maltreatment, especially sexual abuse, in a large-scale questionnaire survey, we do not evaluate child maltreatment. Moreover, gender inequality is considered to be a minor problem since the social status of women has been greatly enhanced in China; therefore, we also do not assess gender discrimination. The participants indicated whether a series of life events had happened to them in the past year. If yes, then they described the degree of tension—mild, medium, strong, or very strong—that the life events caused. The responses were made on a scale that ranged from 1 (never happens), 2(mild) to 5 (very strong).

1. Health pressure. The 9-item health pressure subscale of the Stressful Life Events Scale [39] was used (e.g., “I suffered from illness”). The reliability of this subscale in this study was Cronbach’s *α* = 0.812. The mean score was also calculated. The participants whose percentile was equal to or greater than 75% were coded as 1 (risk exposure); the other participants were coded as 0 (no risk). The 75th percentile cut-off was used (1 = score above the 75th percentile). This cut-off point has been used in other risk studies [8–12].
2. Family economic pressure. This subscale consisted of the three items of family poverty, housing purchase and debt. The reliability of this subscale in this study was Cronbach’s *α* = 0.745. The mean score was also calculated. The participants whose percentile was equal to or greater than 75% were coded as 1 (risk exposure); the other participants were coded as 0 (no risk).
3. Love and marriage problems. The 6-item love and marriage problem subscale from the Stressful Life Events Scale [39] was employed (e.g., “I didn’t get along with my partner”). The reliability of this subscale in this study was Cronbach’s *α* = 0.842. The mean score was also calculated. The participants whose percentile was equal to or greater than 75% were coded as 1 (risk exposure); the other participants were coded as 0 (no risk).
4. Conflicts among family members. Conflicts among family members was measured by the 6-item conflicts among family members subscale from the Stressful Life Events Scale [39] (e.g., “I didn’t get along with my parents”). The reliability of this subscale in this study was Cronbach’s *α* = 0.821. The mean score was also calculated. The participants whose percentile was equal to or greater than 75% were coded as 1 (risk exposure); the other participants were coded as 0 (no risk).
5. Work stress. Work stress was assessed by the 23-item Work Stress Scale [40, 41], which was developed based on the Source of Pressure Subscale from the Occupational Stress Indicator Questionnaire [42] and consisted of the following six dimensions: working conditions and workload (e.g., “I often have to work overtime”); role stress (e.g., “my job responsibility is ambiguous”); relationships with others (e.g., “I receive little support from my superior”); organizational structure and climate (e.g., “There are unreasonable policies, regulations and procedures at my organization”); home-work interface (e.g., “work and taking care of my family put me under great pressure”); and career and achievement (e.g., “I have little hope of promotion”). The reliability of this scale in this study was Cronbach’s *α* = 0.857. The mean score was also calculated. The participants whose percentile was equal to or greater than 75% were coded as 1 (risk exposure); the other participants were coded as 0 (no risk).
6. Friend support. The 4-item friend support subscale from the Perceived Social Support Scale [43] was used, whose Chinese version was revised by Huang, Jiang and Ren (1996) [44]. Example items were “My friends really try to help me” and “I can count on my friends when things go wrong”. The responses were provided on a scale that ranges from 1 (strongly disagree) to 5 (strongly agree). The reliability of this subscale in this study was Cronbach’s *α* = 0.820. The mean score was also calculated. The participants whose percentile was equal to or less than 25% were coded as 1 (risk exposure); the other participants were coded as 0 (no risk).

The agentic stressor index was obtained by adding the family economic pressure index and the work stress index, the communal stressor index was obtained by adding the indices of love and marriage problems, conflicts among family members, and friend support, and the cumulative risk index was obtained by adding the aforementioned six risk indices.

#### Problem-focused coping

Problem-focused coping was measured by three dimensions—active coping, planning, and using instrumental support—from the Brief COPE scale [45] with 6 items. Example items were “I have been taking actions to try to make the situation better” and “I have been thinking hard about what steps to take”. The English version was translated into Chinese by two psychologists and one English doctor and was then back-translated by another English doctor. This translation and back-translation process was repeated before the final form was established. The responses were made on a 5-point scale that indicates the frequency with which the participants used the coping styles in daily life, which ranged from 1 (seldom) to 5 (always). The results of the confirmatory factor analysis were χ^2^/df = 3.56, CFI = 0.932, TLI = 0.910, and RMSEA = 0.049, which showed that the fit indices all met the cut-off criteria. The reliability of the three dimensions for the Chinese version used in this study was *α* = 0.869.

#### Depression

The 20-item Self-rating Depression Scale (SDS) [46] was used in this study, whose Chinese version was revised by Wang and Chi (1984) [47]. Example items were “I feel depressed” and “I am hopeful about my future”. The responses were made on a 4-point scale that indicated the frequency with which the participants experienced symptoms in the last week, which ranged from 1 (never or seldom) to 5 (often or always). The reliability of the scale for the Chinese version used in this study was *α* = 0.812. A standard score was obtained by multiplying the raw score by 1.25 and then by taking the resulting integer.

### Statistical method

We used two approaches to measure the gender differences in depression: Odds ratio (OR) of depression between women and men, and the mean differences of depression between male and female employees. Correlation coefficients difference test was applied to examine which factor was more related to depression for male and female employees, and moderating effect test was used to examine the gender differences in the interaction effect of cumulative risk and problem-focused coping on depression.

#### Odds ratio (OR)

The odds ratio (OR) evaluates whether the odds of a certain outcome (e.g., depression) is the same for two groups (e.g., men and women). For the gender differences in depression, the OR values are equal to ratio of the odds of depression among women (the number of depressed women divided by the number of non-depressed women) to the odds of depression among men (the number of depressed men divided by the number of non-depressed men). Thus, values greater than 1 indicate that women have greater odds of depression compared with men.

#### Correlation coefficients difference test

The correlation coefficients (r) are converted to Zr according to the r-Zr Conversion Table [48]. Then, the difference test statistic for the correlation coefficient between two independent samples is calculated according to the following formula:

$$Z=(Zr_{1}-Zr_{2})/\sqrt{1/(n_{1}-3)+1/(n_{2}-3)}$$

#### Moderating effect test

The moderating effect test is based on the following steps. First, multicollinearity testing is conducted. Regression analysis is used to model depression as a function of cumulative risk and problem-focused coping while controlling for age, education, and position. Tolerances greater than 0.1 or variance inflation factors (VIF) less than 10 indicate that multicollinearity is not a serious problem. Second, the data are split into two parts according to gender. Third, a hierarchical regression analysis is conducted. Age, education, and position are entered as the control variables, cumulative risk and problem-focused coping are entered as predictors, and the interaction term of cumulative risk and problem-focused coping is entered to examine the interaction/moderation effects. A significant standardized regression coefficient for the interaction term indicates a significant moderation effect [49]. Fourth, if the standardized regression coefficient of the interaction term is significant, then the range of the significance of the moderator is calculated by the Johnson-Neyman method [50–52]. The significance level is set at *α* = 0.05, and the range of significance of the moderator is calculated according to the SPSS procedure described by Hayes and Matthes (2009) [50]. Fifth, the moderating effect is illustrated by using one-standard deviation offsets of the independent variable and moderator. In addition, one value of the moderator should be within the range of significance of the moderator, and the other value should be outside the range. The abovementioned four values are substituted into the regression equation, and a line chart of the moderating effect is drawn.

## Results

### Gender differences in cumulative risks, problem-focused coping and depression

Table 1 shows the gender differences in the stressors, problem-focused coping and depression among adult employees. As far as the stressors are concerned, the agentic stressor of male employees was significantly higher than that of female employees (*t* = 3.10, *p* < 0.01, d = 0.31), there was no gender difference in the communal stressor (*t* = 0.51, *p* > 0.05), and the cumulative risk of male employees was marginally significantly higher than that of female employees (t = 1.84, *p* = 0.067, d = 0.19). There was no significant difference in the utilization frequency of problem-focused coping between male and female employees. The level of depression of male employees was significantly lower than that of female employees (*t* = -3.11, *p* < 0.01, d = 0.32). OR = (107/63)/(115/121) = 1.79.

**Table 1:** Gender differences in stressors, problem-focused coping and depression among adult employees.

Variables	Male			Female			t	d
	M	SD	n	M	SD	n		
health pressure	0.26	0.44	236	0.24	0.43	170	0.49	0.05
agentic stressor	0.65	0.78	236	0.42	0.68	170	3.10**	0.31
communal stressor	1.26	0.78	236	1.22	0.81	170	0.51	0.05
cumulative risk	2.17	1.55	236	1.88	1.55	170	1.84+	0.19
problem-focused coping	2.97	0.75	236	2.91	0.85	170	0.71	0.07
Depression	52.7	9.62	236	55.59	8.63	170	-3.11**	0.32

n = 406,

^+^ *p* < 0.08,

* *p* < 0.05,

** *p* < 0.01

### Gender differences in the correlation coefficients

The gender differences in the correlation among the stressors, problem-focused coping and depression among adult employees are shown in Table 2. It can be observed that there were no gender differences in the correlation coefficients between depression and the stressors (health pressure, agentic stressor, communal stressor, and cumulative risk). Depression was significantly negatively related to problem-focused coping for both male and female employees. However, the correlation coefficient for male employees (r = -0.456, *p* < 0.01) was significantly greater than the correlation coefficient for female employees (r = -0.217, *p* < 0.01, Z = 2.69, *p* < 0.01).

**Table 2:** Gender differences in the correlation among stressors, problem-focused coping and depression among adult employees.

Variables	Male	Female	Z
	Depression	Depression	
health pressure	0.260**	0.109	1.54
agentic stressor	0.227**	0.182*	0.46
communal stressor	0.048	0.084	-0.36
cumulative risk	0.211**	0.154*	0.56
problem-focused coping	-0.456**	-0.217**	2.69**

n = 406,

* *p* < 0.05,

** *p* < 0.01

### Gender differences in the moderating effect

Multicollinearity testing showed that the minimum tolerance was 0.698, and the maximum VIF was 1.433, which indicated that multicollinearity was not a serious problem. All the data were used to conduct a hierarchical regression analysis. As shown in Table 3, the interaction term of cumulative risk and problem-focused coping was significant (t = -2.611, *p* < 0.01), which indicated that the moderating effect was significant and that the hypothesis was validated.

**Table 3:** Moderating effect of problem-focused coping on cumulative risk and depression among adult employees.

Variables	B	*β*	SE	t	F
constant	58.52		3.67	15.96***	16.78***
gender	1.85	0.10	0.89	2.06*	
age	-0.10	-0.05	0.10	-1.00	
education	-1.93	-0.17	0.57	-3.35**	
position	2.94	0.11	1.21	2.42*	
Cumu_risk	4.06	0.68	1.09	3.73***	
Pro_cop	-2.14	-0.18	0.85	-2.53*	
Cumu_risk*Pro_cop	-0.93	-0.50	0.36	-2.61**	

Cumu_risk: cumulative risk, Pro-cop: problem-focused coping,

* *p* < 0.05,

** *p* < 0.01,

*** *p* < 0.001

Then, the data were divided into two groups according to gender. The results of the moderating effect test for the male and female employees are shown in Table 4. The standardized regression coefficient for the interaction term of cumulative risk and problem-focused coping was significant for male employees (*t* = -2.04, *p* < 0.05), which showed that the moderating effect for male employees was significant. However, the standardized regression coefficient for the interaction term was not significant for female employees (*t* = -0.76, *p* > 0.05), which indicated that the moderating effect for female employees was not significant.

**Table 4:** Gender differences in the moderating effect of problem-focused coping on cumulative risk and depression among adult employees.

Gender	Variables	B	*β*	SE	t
Male	Control variables				
	constant	67.01		4.76	14.07***
	age	-0.29	-0.15	0.13	-2.24*
	education	-0.90	-0.07	0.76	-1.19
	position	3.60	0.12	1.76	2.04*
	Independent variables				
	Cumu_risk	4.14	0.68	1.45	2.86**
	Pro_cop	-3.83	-0.3	1.24	-3.09**
	Interaction term				
	Cumu_risk*Pro_cop	-0.93	-0.47	0.48	-2.14*
Female	Control variables				
	constant	57.96		4.45	13.04***
	age	0.10	0.06	0.14	0.71
	education	-3.53	-0.34	0.86	-4.13***
	position	3.05	0.14	1.64	1.86
	Independent variables				
	Cumu_risk	2.29	0.41	1.66	1.38
	Pro_cop	-1.17	-0.11	1.14	-1.03
	Interaction term				
	Cumu_risk*Pro_cop	-0.40	-0.24	0.53	-0.76

Cumu_risk: cumulative risk, Pro-cop: problem-focused coping,

* *p* < 0.05,

** *p* < 0.01,

*** *p* < 0.001

**Fig 1:**
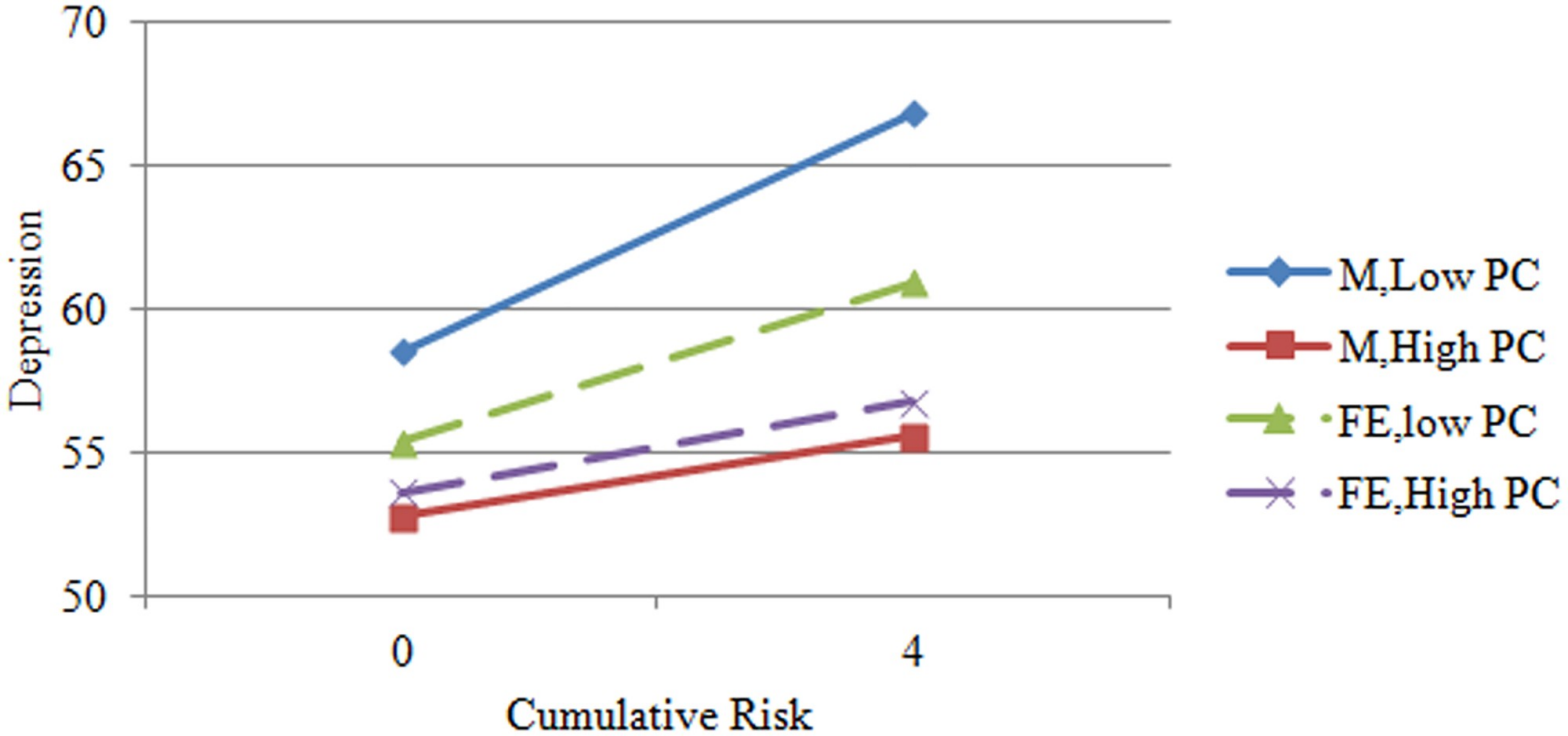
Gender differences in the moderating effect of problem-focused coping on cumulative risk and depression among adult employees. Note: M: Male, FE: Female, PC: Problem-focused Coping.

The moderating effect is illustrated below. First, we applied the values of cumulative risk and problem-focused coping. The mean and standard deviation of the cumulative risk for male employees were 2.17±1.55, and the value of cumulative risk was set to an integer; thus, the values of 0 (less than one standard deviation below the mean) and 4 (greater than one standard deviation above the mean) were taken. The mean and standard deviation of problem-focused coping for male employees were 2.97±0.75. The significance range of the moderator (problem-focused coping) for male employees was [1.00, 3.38] according to the Johnson-Neyman method. The value of 2.22 (one standard deviation below the mean) within the significance range of the moderator and 3.72 (one standard deviation above the mean) outside the significance range of the moderator were taken for problem-focused coping. The four aforementioned values were entered into the regression equation for male employees, and a line chart depicting the moderating effect was drawn (Fig 1). The aforementioned four values were also entered into the regression equation for female employees, and corresponding lines illustrating the moderating effect were added to Fig 1.

## Discussion

### Gender differences in stressors, problem-focused coping and depression

The first purpose of this study is to examine the gender differences in the stressors among adult employees. It can be seen from Table 1 that the agentic stressor of male employees was significantly greater than that of female employees, which is similar to adolescent. Male employees are expected to buy houses and earn money to support their families in China, while female employees are expected to just have a job, thus their agentic stressor is significantly different. However, unlike teenagers, there was no significant difference in the communal stressor between male and female employees. With the development of Chinese society, men and women are becoming increasingly more equal. Many men are very concerned about their relationship with their wife. To maintain their relationship with their wife, many men give their salary cards to their wife and perform housework after returning home. They also help their children with their homework and send them to and from school. In addition, many male employees have to address their relationships with their friends, leaders and customers for their career development. Therefore, the communal stressor was similar between male and female employees.

The second purpose of this study is to investigate the gender differences in the utilization frequency of problem-focused coping among adult employees. The participants were young (the mean age was 25.9 years old) in metropolitan and received a good education (78.1% of them completed higher education). Mean difference testing showed that there were no gender differences in the utilization frequency of problem-focused coping (Table 1), which was consistent with the results from Kaliterna et al. (2009) [53] and Monteiro et al. (2014) [54]. These young employees from economically developed areas had received a good education and were busy with work. They had to keep solving various problems that pertained to learning, examinations, job-hunting, work, money-making, promotions, etc., and had formed the habit of using a problem-focused coping style when faced with stressful events. Therefore, there was no significant difference between male and female employees regarding the utilization frequency of problem-focused coping. The popularity of the word "manly woman", which expresses the characteristics of contemporary Chinese women’s independence, self-reliance, self-confidence and optimism, is a reflection of this phenomenon [55].

The odds ratio of the gender differences in depression in this study (1.79), similar to the odds ratio for adults in previous studies (e.g., Salk et al., 2017 [5]), was lower than the odds ratio for adolescents, which might be explained in two ways. On the one hand, girls suffered from more pressures, especially interpersonal pressures [3, 4, 15], whereas the cumulative risks of male employees are marginally significantly higher than that of female employees. On the other hand, boys use more problem-focused coping than girls, while female employees employ increasingly more problem-focused coping that is not significantly different from male employees.

The third purpose of this study is to explore the gender differences in the interaction effect of cumulative risk and problem-focused coping on depression among adult employees through a moderation model. It can be seen in Table 2 that depression was more closely related to problem-focused coping for male employees than for female employees. Problem-focused coping moderated the relationship between cumulative risk and depression for male employees but did not moderate this relationship for female employees (Table 4), that is, higher level of problem-focused coping is associated with a stronger attenuation of the influence of high cumulative risk on depression levels in males than females. As shown in Fig 1, when the cumulative risk was low, the coping requirement was also low; therefore, the levels of depression of the participants were all low and did not significantly differ. However, when the cumulative risk was high, the situation was different. According to gender role socialization theory, men are expected to be independent, aggressive and successful [15, 17]. Male employees with high-frequency problem-focused coping have an enterprising spirit, accept what has happened, make clear plans, actively seek social support, and bravely overcome difficulties. They are more likely to solve problems, realize goals, achieve success, and earn higher social appraisals; thus, their depression levels are relatively low. However, male employees with low-frequency problem-focused coping refuse to accept what has happened, face difficulties with fear, and cannot make clear plans or seriously carry them out. They have a difficult time with receiving social support from other people, solving problems, realizing goals, and achieving success. They are often looked down on by other people and feel inferior and desperate; thus, their level of depression is high.

In contrast, women are generally expected not to be aggressive and achieve success but rather to be gentle and take care of their families. When female employees with high-frequency problem-focused coping solve problems, realize goals, and achieve success, they are often not highly appreciated. For example, the titles of “strong women” and “women doctors” earn approval and praise because they are financially independent, mature, decisive and capable; meanwhile, these women are dispraised for lacking mildness and sacrificing family responsibility. However, when female employees with low-frequency problem-focused coping cannot solve problems, realize their goals, and achieve success, they are unlikely to receive too much criticism or blame; thus, their level of depression is not very high. Accordingly, problem-focused coping has a stronger effect on depression for male employees than for female employees because of the gender differences in social expectations.

### Highlights of this study

Several highlights of our study should be noted. First, cumulative risk is used to evaluate the stressors that lead to depression. Cumulative risk can assess the stressors more comprehensively and accurately than a single stressor and is more parsimonious than the summary score used in previous studies. Second, this study investigates whether adult employees have different characteristics compared with adolescents in terms of stressors and the utilization frequency of problem-focused coping. The stressors of male employees are increasing, whereas female employees increasingly use more problem-focused coping, which may account for the decrease of the gender differences in depression among adult employees. Third, this study explores the gender differences in the interaction effect of cumulative risk and problem-focused coping on depression among adult employees through a moderation model. Compared with previous studies that used correlation and regression analyses to explore the gender differences in the main effect of stressors or problem-solving coping on depression, this study further reveals the gender differences in depression.

### Practical implications

This study has important practical significance for individuals, especially for men when coping with stress. First of all, ones should embrace the spirit of enterprise and keep endeavouring to forge ahead in adversity. They should accept what has happened, make clear plans, actively seek social support, bravely get over one’s difficulties, solve various problem in life, and achieve success to decrease the incidence of depression and lower the risk of suicide. Moreover, cognitive behavioural program can also be used to cope with stress. For example, Williams Life Skill Training which built eight approaches to better identify one’s feelings, communicate with others and solve problems was used and effectively decrease depression and anxiety [56]. In addition, employees should take different coping strategies to different stressors. For instance, ones should give full play to their own strengths, improve their own capabilities, create more value for society, and increase their economic income when they are faced with economic pressure. They should learn some interpersonal communication skills such as empathy, praise, conflict management, make use of some interpersonal rules such as Primacy and Recency effects, fairness and social exchange, and improve interpersonal relationship. To effectively cope with work stress, they should improve their work competence and efficiency, try to obtain understanding and support from the leaders. They should also develop good living habits, properly do exercise, adjust diet, and keep a good attitude in face of health problem. Last but not the least, employers should attempt to encourage their employees to fight against their difficulties for the good of the employees and the enterprises.

### Limitations and future directions

Despite its contributions, this study has several limitations that should be acknowledged. The first limitation is that the sample only consisted of employees aged from 18 to 42 in two metropolitan cities in China. Since more and more Chinese have received higher education and worked in cities with the development of education and urbanization in China, the participants in this study might well represent the population of young and middle-aged employees. However, this study did not investigate the employees who were older than 42 years and might have different characteristics. Future research should use more diverse samples to demonstrate whether the results from this study can be generalized to other samples. The second limitation is that we only made investigation in China in collectivistic culture. Do adults employees in individualistic culture have similar characteristics? Future research should examine the generalizability in other culture. The third limitation concerns the self-report nature of the data, which may be subject to a social desirability effect that might include responses that are subjective and inaccurate. Future research should use more methods, such as ratings from other people, to assess problem-focused coping and depression.

## Conclusion

In conclusion, unlike adolescence, there was no gender difference in communal stressors, and the cumulative risk of male employees was marginally significantly higher than that of female employees. There was no significant difference in the utilization frequency of problem-focused coping between male and female employees. The stressors of male employees are increasing, while female employees increasingly use more problem-focused coping, which may account for the decrease of the gender differences in depression for adults.

The gender differences in depression among adult employees were influenced by the interaction of cumulative risk and problem-focused coping. Problem-focused coping moderated the relationship between cumulative risk and depression for male employees but not for female employees. For male employees with high-frequency problem-focused coping, there was no significant difference in depression under low and high cumulative risk, but for male employees with low-frequency problem-focused coping, there was significant difference in depression under low and high cumulative risk. Problem-focused coping has a stronger effect on depression for male employees than for female employees because society expects men to be independent, enterprising and successful. People, especially men, should keep endeavouring to forge ahead in their life.

## Supporting information

S1 Data The original data of this study with the SPSS files. (ZIP)
